# Basic Psychological Needs Satisfaction Mediates the Association between Self-Control Skills and Subjective Well-Being

**DOI:** 10.3389/fpsyg.2017.00936

**Published:** 2017-06-07

**Authors:** Hod Orkibi, Tammie Ronen

**Affiliations:** ^1^School of Creative Arts Therapies, University of HaifaHaifa, Israel; ^2^Renata Adler Memorial Research Center for Child Welfare and Protection, Gershon Gordon Faculty of Social Sciences, Tel-Aviv UniversityTel-Aviv, Israel

**Keywords:** self-control, subjective well-being, school satisfaction, positivity ratio, self-determination theory, basic needs satisfaction

## Abstract

Although studies have shown that self-control skills (SCSs) are positively linked to both personal and interpersonal outcomes in adolescent students, studies on the putative mechanisms underlying this relationship are scarce. Drawing on Self-Determination Theory and previous studies, we theorized that the association between students’ SCSs and their subjective well-being (SWB) in school may be mediated by students’ perceived satisfaction of their basic psychological needs for competence, relatedness, and autonomy. The sample consisted of 1576 Israeli adolescent students (54% girls) in grades 10–12 (mean age 16) enrolled in 20 schools. A mediation model was tested with structural equation modeling and a robust bootstrap method for testing indirect effects, controlling for school-level variance. The findings supported the hypothesized model and a *post hoc* multi-group comparison analysis yielded gender invariance in the model. The findings suggest that both girls and boys with high SCSs may perceive themselves as having greater needs satisfaction in school and consequently higher school-related SWB. Implications for policy and practice are discussed.

## Introduction

Adolescents spend most of their time in school during a developmental period that is often associated with increased stress and turbulent emotional experiences (Colten and Gore, 1991; Silvers et al., 2012). Thus, promoting school-related subjective well-being (SWB) is crucial. Within a positive psychological framework, global or general SWB consists of two indicators: cognitive evaluation of life as satisfying and the experience of more frequent positive emotions than negative emotions (Diener et al., 1999, 2009). Most studies have examined global or general SWB in adults but only a few studies on adolescents have examined both the cognitive and emotional indicators of SWB in school. However, focusing on adolescent students’ SWB in school is important because it may differ from their assessment of their general or other domain-specific SWB (Huebner et al., 2000; Zappulla et al., 2014). The accepted conceptualization of SWB in school consists of school satisfaction and the experience of more frequent positive emotions than negative emotions in school (Long et al., 2012; Liu et al., 2016; Tian et al., 2016).

The current study explored the contribution of self-control skills (SCSs), which have been linked to general SWB in both adults and adolescents (Hamama et al., 2012; Orkibi et al., 2014), to students’ SWB in school.

Drawing on Self-Determination Theory, which is one of the major theories related to well-being that has been empirically applied to the school context (Niemiec and Ryan, 2009; Tian et al., 2014a), we theorized and tested a model with students’ perceived basic psychological needs satisfaction in school as a mediator accounting for this link. Studying the direct and indirect links between SCSs and SWB in school are important not only for a better understanding students’ processes in an often turbulent developmental period, but can also inform the development and implementation of psycho-educational interventions.

### The Benefits of SWB

Subjective well-being is particularly pertinent in adolescence because this transitional period of intense psycho-physical development can be one of the most difficult phases in life for some individuals (Steinberg, 2013). Studies have shown that students with higher *general* life satisfaction, the cognitive indicator of general SWB, reported better academic achievement, more school-satisfaction and positive school experiences, better relationships with peers and parents, higher self-esteem, levels of hope and sense of meaning, and less personal distress such as anxiety and depression (Gilman and Huebner, 2006; Marques et al., 2011; Nadeau et al., 2015).

Although there is ample evidence for the benefits of positive emotions in adults (for a review see Lyubomirsky et al., 2005; Fredrickson, 2013), less research has been done on the benefits of positive emotions in children and adolescents beyond the role of low positive emotions in developmental psychopathology (for a review see Davis and Suveg, 2014) and negative academic and behavioral outcomes (Roeser, 2001; Cohen, 2006). Research has shown that positive emotions linked positively with school satisfaction, adaptive coping, and student engagement (Lewis et al., 2009; Long et al., 2012; Liu et al., 2015; Orkibi and Tuaf, 2016).

In Israel, studies have consistently shown that adolescents’ positive emotions (not school-specific) were significantly linked to more SCSs and subjective happiness, as well as less hostility and physical aggression (Agbaria et al., 2015; Gavriel-Fried et al., 2015). Israeli adolescents who reported a high positivity ratio – the experience of more frequent positive emotions (e.g., joy, pride, love) than negative emotions (e.g., fear, shame, anger) – also reported higher SCSs and perceived social support (Orkibi et al., 2014, 2015). A high positivity ratio in Israeli adolescents was significantly positively linked to general life satisfaction, past positive and future time perspectives, and negatively linked to past negative and present fatalistic time perspectives (Orkibi, 2015; Orkibi and Dafner, 2015). Israeli adolescent students’ positivity ratio was significantly and positively linked to SCSs and pro-environmental behavior (Kerret et al., 2016). Based on these findings, the current study focused on the ways in which SCSs may contribute to adolescent students’ SWB in school.

### SWB in School

Subjective well-being in school consists of school satisfaction and the experience of more frequent positive emotions than negative emotions in school. The literature on SWB in schools has mostly focused on the cognitive component, namely school satisfaction. In the United States, students who were high on school satisfaction also scored significantly higher on measures of general life satisfaction, hope, and internal locus of control (Huebner and Gilman, 2006). Good teacher-student relationships and perceived peer social support (Jiang et al., 2013) as well as better in-school behavior (Suldo et al., 2014) also significantly positively correlated with school satisfaction.

Fewer studies have examined the cognitive and emotional indicators of SWB in school simultaneously; in other words, both school satisfaction and emotions experienced in school. Generally, school satisfaction has been shown to be significantly positively linked with positive emotions in school and negatively linked with negative emotions in school (e.g., Long et al., 2012). In an extensive line of studies with Chinese adolescent students, SWB in school was generally significantly linked to perceived social support (Tian et al., 2013; Liu et al., 2016), scholastic competence and social acceptance (Tian et al., 2015c). Similarly, SWB in school predicted students’ sense of school belonging (Tian et al., 2015b). In a different study with Chinese adolescent students, a significant bi-directional association was found between basic psychological needs satisfaction at school (i.e., the need for autonomy, relatedness, and competence at school) and students’ SWB in school (Tian et al., 2014a). School satisfaction and positive emotions in elementary school were significantly linked to gratitude and pro-social behaviors of Chinese students (Tian et al., 2015a). Thus overall, an emerging body of evidence points to the importance of SWB in school and its significant link to adaptive intrapersonal and interpersonal outcomes.

### Self-Control Skills (SCS) and SWB

The current study draws on Rosenbaum’s (1990) conceptualization of self-control as a learned repertoire of goal-directed skills that enable people to cope with distress and overcome difficulties related to maladaptive thoughts, emotions, and behaviors. This repertoire of SCS has strong implications for psycho-educational interventions that have been found to improve students’ functioning (Ronen and Rosenbaum, 2010). A substantial body of research suggests that SCS are positively related to students’ academic competency and performance, independent of general intelligence, cognitive ability, and prior achievements (Matthews et al., 2009; Liew et al., 2010; Zhou et al., 2010; Valiente et al., 2013, 2014). Studies on Israeli adolescents have found significant links between high SCS and fewer negative emotions, as well as more self-efficacy belief, positive emotions, and a higher positivity ratio (Ronen and Seeman, 2007; Orkibi et al., 2014, 2015; Orkibi and Ronen, 2015).

In addition to personal benefits, SCS have also been associated with interpersonal and pro-social outcomes. Studies have consistently shown that students with high SCS report higher perceived social support than students with low SCS (Orkibi and Ronen, 2015; Orkibi et al., 2015). Consistent with this trend, students with high SCS also reported less hostile attribution bias (i.e., interpreting others’ intentions or behaviors as hostile and threatening) and less physically aggressive behavior (Hamama and Ronen-Shenhav, 2012; Agbaria et al., 2015; Gavriel-Fried et al., 2015).

Overall, based on the literature, we theorized that students’ SCS would link directly to students’ SWB in school as well as be mediated through students’ perceived satisfaction of their basic psychological needs (Ryan and Deci, 2000). Specifically, we theorized that because SCS include the ability to volitionally exert control over emotions, thoughts, and behaviors, a higher SCS would contribute directly to SWB in school by regulating cognitions, emotions, and behaviors. We also theorized that SCS would link to SWB indirectly, through the contribution of SCS to students’ experiences of autonomy, relatedness, and competence needs satisfaction in school.

### Basic Psychological Needs Satisfaction as a Mediator

Basic psychological needs theory is a sub-theory of a human motivation macro-theory known as Self-Determination Theory (Ryan and Deci, 2000). This theory posits that the satisfaction of the needs for autonomy, relatedness, and competence is crucial for motivation, optimal development, effective functioning, and good health (Milyavskaya and Koestner, 2011). From a general (not domain-specific) perspective, the need for autonomy refers to the need to experience one’s behavior as volitional and self-endorsed rather than as pressured or coerced by forces perceived to be alien to the self. Note that “autonomy literally means ‘self-governing’ and implies, therefore, the experience of regulation by the self” (Ryan and Deci, 2004, p. 451). This is a key point in our proposed link between SCS and needs satisfaction, in that self-regulation is viewed as “an organizational function that ‘coordinates’ systemic behaviors and serves as a foundation for autonomy and the sense of self” (Shogren et al., 2015, p. 257). The need for relatedness refers to the need to feel significant, connected to, and cared for by important others rather than isolated or disconnected from others. The need for competence refers to the need to experience efficacy, mastery, and skillfulness rather than incompetence. The benefits of these needs satisfaction are documented in research across nations, cultures, and many life domains including education, work, healthcare, sport, parenting, and close relationships (see Deci and Ryan, 2008; Milyavskaya and Koestner, 2011).

Among the studies within the educational context, perceived goal mastery and teacher and peer support were significantly linked to school engagement and hope, whereas perceived autonomy was also linked to academic achievement in middle and high school students in the United States (Van Ryzin, 2011). In adolescent soccer (i.e., football) players in the United Kingdom, perceived coach-autonomy support and satisfaction of basic needs were positively linked to vitality and negatively to perceived exhaustion in soccer (Adie et al., 2012). In a study with Chinese adolescents, perceived satisfaction of the three basic psychological needs was significantly associated with self-rated autonomy, sense of school connectedness, and sense of scholastic competence (Tian et al., 2014b). In a longitudinal study in China, basic needs satisfaction reduced adolescent students’ anxiety and depression (Yu et al., 2016). A different longitudinal study in China showed that needs satisfaction significantly contributed to the prediction of the cognitive and emotional components of students’ SWB in school (Tian et al., 2014b). Similarly, needs satisfaction in physical education was positively related to experiences of vitality and negatively related to negative emotions among high school students in Hong Kong (Liu and Chung, 2014).

There are fewer studies on the mediating role of basic needs satisfaction compared to the numerous studies on the direct links between needs satisfaction and outcomes. For example, needs satisfaction mediated the link between community-related self-esteem and well-being in adults in an urban community in the United States (Molix and Nichols, 2013). Needs satisfaction also mediated the inverse association between socioeconomic status (SES) and physical and mental health among adults in the United States (González et al., 2016). Competence and relatedness needs satisfaction mediated the link between coach-autonomy support and subjective vitality over two seasons in adolescent soccer players in the United Kingdom (Adie et al., 2012). In samples of adults in India and the United States, the relationships between perceived capabilities (i.e., personal, social, and material conditions) and both general SWB and the vitality-meaning composite score were mediated by basic needs satisfaction (DeHaan et al., 2015). In another study that examined multiple mediators in a sample of Chinese adolescent students, relatedness and competence needs satisfaction at school mediated the link between gratitude and SWB in school; autonomy needs satisfaction mediated the link between relatedness and competence needs satisfaction and SWB in school (Tian et al., 2016).

Causal Agency Theory, which emerged in the field of special education and is an extension of the functional model of self-determination (see Shogren et al., 2015, 2017) is particularly germane to the present study and the proposed link between SCS and needs satisfaction. Briefly, this theory posits that the motivation to satisfy basic psychological needs drives people to self-determined (self-caused) actions that enable them to act as causal agents in their own lives, thus enhancing self-determination and overall well-being. Self-determined actions, that lead to causal agency, are characterized by being volitional (i.e., consciously and autonomously self-initiated), agentic (i.e., self-directed toward a goal), and driven by action-control beliefs (i.e., one’s beliefs about the relationships between actions/means and ends). Importantly, self-determined actions are “self-regulated and self-directed… [enabling] a person to make progress toward freely chosen goals and to respond to opportunities and challenges in their environments” (Shogren et al., 2015, p. 259). This is consistent with the abovementioned link between self-regulation, a sense of autonomy and a sense of self.

In line with this view, we reasoned that because SCS are goal-directed skills that help people regulate their emotions, cognitions and behaviors (Rosenbaum and Ronen, 2013), SCS should in turn lead to greater self-determination in terms of helping students experience a greater sense of *autonomy*, volition, and self-endorsement of their behavior in school as well as a sense of *relatedness*, belonging, and genuine connection with teachers and peers, and a sense of *competence* by enabling them to effectively interact with their school environment and maximize opportunities to express or develop their capabilities and strengths.

### Study Hypotheses and Model

Three hypotheses were formulated and tested. First, drawing on previously established links between SCS and general SWB in both adults and adolescents (e.g., Hamama et al., 2012; Orkibi et al., 2014), Hypothesis 1 posited that SCS would positively and directly link to the school-related positivity ratio and school satisfaction – the emotional and cognitive components of SWB in school, respectively. Based on the association in Causal Agency Theory between self-regulation and self-determination (Shogren et al., 2015; Wehmeyer and Shogren, 2017), Hypothesis 2 posited that SCS would be positively related to perceived basic psychological needs satisfaction in school, which reflects self-determination (Niemiec and Ryan, 2009). Finally, the current study contributes to the literature by focusing on the processes underlying the link between students’ SCS and students’ SWB in school. Drawing on previously established links between basic psychological needs satisfaction in school and SWB in school (e.g., Tian et al., 2014a), Hypothesis 3 posited that the link between SCS and SWB in school would be mediated by basic psychological needs satisfaction in school.

## Materials and Methods

### Sample

The study sample was composed of 1576 adolescents (54% girls) in grades 10–12, aged 14–18 (*M* = 16, *SD* = 0.88) enrolled in 20 typical public schools located in the northern, central, and southern regions of Israel. Of this sample, 77% were born in Israel, 12% in the former Soviet Union, and 11% in “other” countries. Eighty-five percent of the sample had married parents, 12% divorced or separated parents, 2% a single parent, and 1% had “another” family situation. Most students (53%) were from a three-child family and were the eldest (48%). Most students (60%) stated their family’s financial situation was “similar” to that of their peers.

### Procedure

Permission to conduct the study was obtained from the Israel Ministry of Education and the University’s Institutional Review Board. The students’ parents received a printed letter informing them of the study and were asked their permission to allow their child to complete the questionnaires. Parents were provided with ample time to respond in writing or by telephone. To protect students from feeling coerced into participation and to prevent any potential for coercion and/or undue influence, it was clarified that participation in the study was on a voluntary basis. It also was explicitly explained to the students that they had the right to refuse to participate or to withdraw from the study at any time, without any penalty or prejudice to their interests. Data were collected using internet-based survey software where logging-in to the online questionnaire signified student assent. Students responded during regular school hours in computer classrooms, proctored by a research assistant. Because the survey software did not let items be skipped, there were no missing data from the participating students.

### Measures

The sociodemographic questionnaire asked students to provide the following information: gender, age, country of birth, grade level, school, parental marital status, number of siblings, and socio-economic status compared to other students in their class.

#### Self-Control Skills

The 32 item Adolescents’ Self-Control scale assessed students’ SCS (Rosenbaum and Ronen, 1991) in coping with disturbing thoughts, emotions and behavior such as solving skills, attentional control (i.e., distraction), cognitive reframing, delay of gratification, and use of self-talk and self-reinforcement. For example: “When I have to do boring homework, I think about how important it is for me.” Students rated the items on a scale ranging from 1 (*not characteristic of me at all)* to 6 (*very characteristic of me*), with higher mean scores indicating higher SCS. The scale’s validity and reliability have been established (Zauszniewski et al., 2010), including its Hebrew version (Ronen and Rosenbaum, 2010; Orkibi et al., 2014). In the present study, the internal consistency coefficient was 0.84. In contrast to scales that measure self-control as a trait, this scale measures cognitive-behavioral skills that are shaped by experience and practice and thus has stronger implications for future psycho-educational interventions (for a review of self-control measures, see Duckworth and Kern, 2011).

#### Perceived Needs Satisfaction at School

The Students’ Basic Psychological Needs at School scale was developed based on Self-Determination Theory (Tian et al., 2014b). This self-report scale consists of 15 items, five items for each subscale: need for autonomy (e.g., “I am free to arrange my studies and extracurricular activities at school”), need for relatedness (e.g., “I get along well with my teachers and classmates at school”), and need for competence (e.g., “I have been able to learn interesting new skills at school recently”). Students rated items on a scale ranging from 1 (*strongly disagree*) to 6 (*strongly agree*). Previous results provide good support for the validity and reliability of the scale (Tian et al., 2014b). In the present study, the internal consistency reliability coefficients of the total score was α = 0.82. Because we were interested in need satisfaction in general, the mean of the 15 items was used in the analysis with higher scores representing greater perceived satisfaction of basic psychological needs.

#### School Satisfaction

It was measured on the 8 item school subscale of the 40 item Multidimensional Students’ Life Satisfaction Scale that covers important life domains (Huebner, 1994; Huebner et al., 1998). Students rated their overall satisfaction with their school experiences on a scale ranging from 1 (*strongly disagree*) to 6 (*strongly agree*), where higher mean scores indicating greater school satisfaction. The scale has demonstrated good validity and reliability (Huebner et al., 1998). In the current study, the internal consistency reliability coefficient of the scale was α = 0.86.

#### The Positive and Negative Affect Scale for Children

It is a 30 item self-report scale with 15 items for positive emotions and 15 items for negative emotions (Laurent et al., 1999). To measure the school-related positivity ratio, students ranked the frequency of each positive emotion (e.g., joy, pride, love) and negative emotion (e.g., fear, shame, anger) they had experienced in the previous few weeks *at school* using a scale ranging from 1 (*very few times*) to 5 (*a lot of time*s). Laurent et al. (1999) reported evidence for good validity and reliability. In the current study, the alphas were α = 0.89 for positive emotions and α = 0.90 for negative emotions. To obtain a *positivity ratio* score, the mean score for positive emotions items was divided by the mean score for negative emotions items (Orkibi et al., 2015). A larger ratio (higher score) thus represented a greater number of positive over negative emotions.

### Data Analysis

First, bivariate correlations for all the variables were explored. Second, we used IBM’s Amos23 for a path analysis to test the theoretical model, particularly the mediating role of psychological needs satisfaction (mean score) in the *indirect* association between SCS (mean score) and school satisfaction (mean score) and the positivity ratio (the ratio of positive versus negative emotions in school). We ran the analyses while controlling for student age and school-level variance in SES as indicated by values provided by the Israeli Ministry of Education. School satisfaction and positivity ratio were allowed to co-vary. The model’s fit to the data was evaluated using the criteria of χ^2^/df ≤ 3, a comparative fit index (CFI) ≥ 0.95, Tucker–Lewis coefficient (TLI) ≥ 0.95, and a root mean square error of approximation (RMSEA) < 0.80 (Schreiber et al., 2006). The bootstrap method for testing indirect effects (i.e., mediation) was used with the confidence level set at 0.95 and bootstrap bias-corrected samples set at 5000. When zero is not in the 95% confidence interval, the indirect effect is significantly different from zero at *p* < 0.05 (two tailed) (Preacher and Hayes, 2004, p. 722). *Post hoc* multiple group comparison analysis was conducted to test whether the model differed by gender in structural weights (i.e., regression weights) using Amos multiple-group automated procedure (Byrne, 2010).

## Results

### Descriptive Statistics

**Table 1** presents the means and standard deviations for SCS, perceived needs satisfaction at school, school satisfaction, and the positivity ratio. For each variable, higher scores indicate higher levels of that psychological construct. As can be seen, of the three psychological needs satisfaction at school, relatedness had the highest score.

**Table 1:** Descriptive statistics and Pearson’s correlations between the variables.

Variables	Self-control skills	Autonomy	Relatedness	Competence	School satisfaction	*M*	*SD*
Self-control skills	(0.84)					25.15	22.45
Autonomy	0.30	(0.80)				3.97	1.02
Relatedness	0.36	0.46	(0.83)			5.02	0.79
Competence	0.40	0.48	0.49	(0.75)		4.25	0.97
School satisfaction	0.42	0.52	0.54	0.61	(0.86)	4.06	0.96
Positivity ratio	0.40	0.39	0.43	0.44	0.47	1.65	0.74

*All correlations were significant at the 0.001 level. Cronbach’s alphas are displayed in parentheses on the diagonal. *N* = 1576*.

### Correlation Analyses

**Table 1** presents inter-correlations between the observed values of all the variables, confirming Hypothesis 1 and Hypothesis 2. Although all the correlations were significant, the strongest positive correlations were between psychological needs satisfaction at school and school satisfaction. Of the three psychological needs, competence had the strongest correlation with school satisfaction.

In addition, Pearson correlation analysis between age and the variables yielded significant, albeit weak, correlations between age and SCS (*r*_s_ = 0.07, *p* < 0.01), autonomy (*r*_s_ = -0.07, *p* < 0.01), competence (*r*_s_ = -0.05, *p* < 0.05), and school satisfaction (*r*_s_ = 0.09, *p* < 0.01). Spearman rho correlations between gender (coded: 1 = boys, 2 = girls) and the variables yielded significant correlations between gender and SCS (*r*_s_ = 0.11, *p* < 0.001) with girls scoring significantly higher (*M* = 27.20, *SD* = 22.90) than boys (*M* = 22.74, *SD* = 21.70); between gender and autonomy (*r*_s_ = -0.08, *p* < 0.01) with boys scoring significantly higher (*M* = 4.06, *SD* = 0.98) than girls (*M* = 3.90, *SD* = 1.05); between gender and school satisfaction (*r*_s_ = 0.07, *p* < 0.01) with girls scoring significantly higher (*M* = 4.11, *SD* = 0.96) than boys (*M* = 3.99, *SD* = 0.96); and between gender and positivity ratio (*r*_s_ = -0.20, *p* < 0.001) with boys scoring significantly higher (*M* = 1.79, *SD* = 0.73) than girls (*M* = 1.53, *SD* = 0.72). Student-reported SES correlated significantly with the positivity ratio in school (*r*_s_ = 0.06, *p* < 0.05). Given these correlations, age, gender, and SES were included in the analyses described below.

### Theorized Mediation Model

Given the significant correlations between age and the other variables, age was included in the model to control for its potential effect on the model variables. Student-reported SES was first included in the model but then omitted because there was no effect on the variables in the model. Importantly, we ran the analyses controlling for school-level variance in SES as indicated by each school’s Nurturing Index score that was provided by the Israeli Ministry of Education. A Nurturing Index score is a variable that ranges from 1 to 10 and reflects the overall SES characterizing a given school’s student body as a whole (see BenDavid-Hadar and Ziderman, 2010). We thus added paths from the school-level SES variable to the model variables.

**FIGURE 1:**
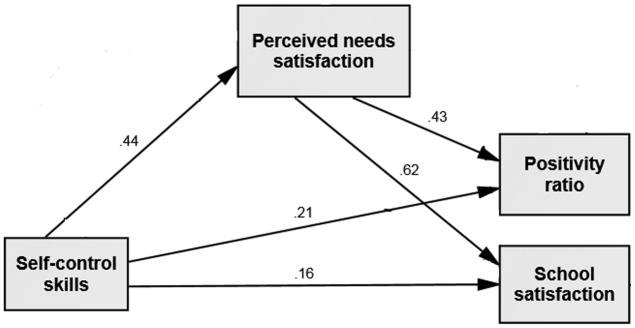
Hypothesized model with perceived needs satisfaction as the mediator between self-control skills and the school related positivity ratio and school satisfaction for the entire sample (*N* = 1576). Standardized regression weights are presented. Covariates and controlled variables (age and school-level SES) are omitted for clarity. All paths were significant at the *p* < 0.01 level.

The path analysis indicated that the theorized model depicted in **Figure 1** provided a good fit to the data as shown in **Table 2**. A significant *indirect* link, through perceived psychological needs satisfaction as a mediator, was found between SCSs and school satisfaction (95% CI = [0.242, 0.301], *p* < 0.01) as well between SCS and the school-related positivity ratio (95% CI = [0.164, 0.215], *p* < 0.01). Because the CI did not include zero, the null hypothesis of no mediation was rejected in both paths. Thus, the findings confirmed the model suggested in Hypothesis 3. Note that the explained variance was 20% for psychological needs satisfaction, 30.5% for positivity ratio, and 50% for school satisfaction.

**Table 2:** Goodness-of-fit indices for the theorized, alternative, and gender models.

Model	χ^2^	*df*	χ^2^/*df*	*p*	CFI	TLI	RMSEA (90% CI)
Theorized	8.15	3	2.72	0.043	0.99	0.99	0.03 [0.005, 0.061]
Alternative 1	9.43	3	3.143	0.024	0.99	0.98	0.04 [0.012, 0.065]
Alternative 2	26.25	4	6.56	0.001	0.99	0.95	0.06 [0.039, 0.052]
Gender constrained	17.24	16	1.08	0.370	0.99	0.99	0.007 [0.000, 0.025]
Gender unconstrained	10.11	6	1.68	0.120	0.99	0.99	0.2 [0.000, 0.042]

*df, degrees of freedom; CFI, comparative fit index; TLI, Tucker–Lewis index; RMSEA, root mean square error of approximation*.

### Alternative Mediation Models

**FIGURE 2:**
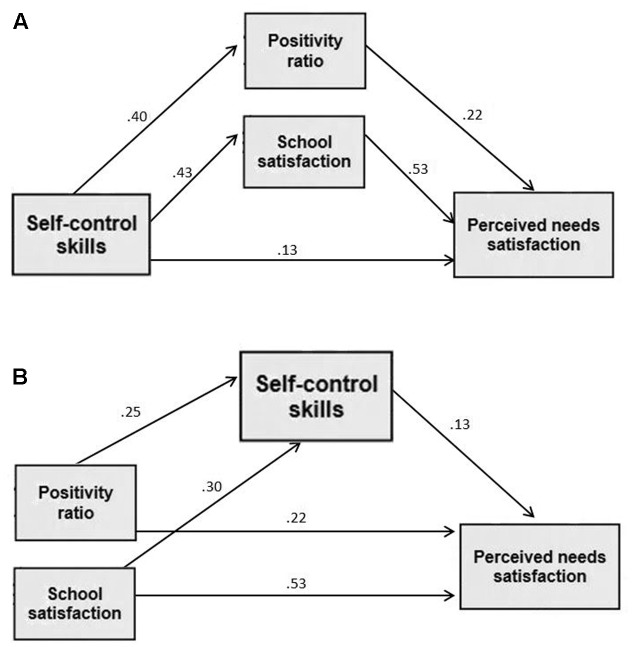
Two alternative models with reverse ordering of the variables. The first alternative model is **(A)** and the second alternative model is **(B)**. Standardized regression weights are presented. Covariates and controlled variables (age and school-level SES) are omitted for clarity (*N* = 1576). All paths were significant at the *p* < 0.01 level.

As advocated in the methodological literature (James et al., 2006), we ran *post hoc* path analyses of two alternative models with reverse ordering of the variables to examine the theoretical possibility of reverse causation. The models were compared in terms of model fit indices. Based on studies showing bidirectional links between SWB and needs satisfaction (Tian et al., 2014a), in the first alternative model, **Figure 2A**, SCS was linked to school related positivity ratio and satisfaction (the mediators), that in turn was linked with psychological needs satisfaction. The second alternative model, **Figure 2B**, was based on Baumeister’s idea that in the presence of positive emotions people can exert better self-control (e.g., Baumeister et al., 2007; Baumeister and Sparks, 2008). Accordingly, school related positivity ratio and satisfaction *preceded* SCS (the mediator), which in turn was linked with perceived needs satisfaction as an outcome. As shown in **Table 2**, the analysis of the two alternative models yielded fit indices that were inferior to those of the hypothesized model.

### Theorized Model Invariance across Gender

Given the abovementioned significant gender-related results we conducted a *post hoc* analysis to test whether the theorized mediation model differed for girls (*n* = 848) versus boys (*n* = 728). As shown in **Table 2**, we constructed two models for comparison: an unconstrained model that posited a distinctive model for each gender group, and a fully constrained model that posited equality (i.e., invariance) on all regression weights between the gender groups (Byrne, 2010). The results of model comparison showed that the constrained and unconstrained models did not significantly differ (Δχ^2^ = 7.132, Δ*df* = 10, *p* = 0.71). A CFI difference (ΔCFI = 0.001) was used as a criterion for the model invariance as recommended by Cheung and Rensvold (2002). A value of CFI less or equal to 0.01 indicates that the null hypothesis of invariance should *not* be rejected. The Akaike information criterion (AIC) which is used for model comparison, with lower values reflecting better fit (Schreiber et al., 2006), also indicated that the constrained model (69.24) was superior to the unconstrained model (82.11). These findings indicate that, overall, the model was *not* significantly different for girls and boys.

## Discussion

Whereas most studies have focused on negative links between SCS and maladaptive outcomes, this study contributes to the growing empirical evidence on the significant positive links between SCS and adaptive outcomes (Orkibi et al., 2014; Gavriel-Fried et al., 2015; Kerret et al., 2016). Specifically, the findings confirm Hypothesis1 that increases in students’ perceived ability to exert SCS are related to their experience of greater school satisfaction and more positive than negative emotions in school. In other words, the findings point to the possibility that experiencing greater SWB in school is, at least to some extent, due to students’ ability to regulate their cognitions, emotions, and behaviors as measured here.

Furthermore, using a relatively large sample of adolescent students, the present study provides additional empirical support for the application of Self-Determination Theory to the educational context (Tian et al., 2014b), and extends the literature on this topic by confirming Hypothesis 2 that SCS and perceived psychological needs satisfaction are positively correlated. More specifically, SCS may enable students to respond more constructively and cope more successfully with challenges in school, and thus enhance their perception of basic needs satisfaction in school. This interpretation coincides with the conceptualization of self-control as a set of goal-directed skills that enables people to volitionally act upon their aims, overcome difficulties, and thus feel more resourceful and capable (Rosenbaum, 1990; Rosenbaum and Ronen, 2013). It is also consistent with the abovementioned Causal Agency Theory, according to which self-regulated, volitional, and agentic actions may foster adolescents overall well-being through needs satisfaction and self-determination (Wehmeyer and Shogren, 2017). Note that the two alternative models we examined had fit indices that were inferior to those of the hypothesized model, pointing to the possibility that SCS are indeed predictors of perceived needs satisfaction and, consequently, SWB in school – thus confirming Hypothesis 3. This contrasts with the inverse view that positive emotions lead to self-control (e.g., Baumeister et al., 2007; Baumeister and Sparks, 2008). Clearly, additional longitudinal and experimental studies are warranted to further clarify these relationships.

Regarding gender differences, despite the correlations between gender and some of the variables, our theorized model was not significantly different for boys and girls. This suggests that the overall contribution of SCS to SWB in school (direct link), as well as the contribution of perceived basic psychological needs satisfaction to the relationship between SCS and SWB in school (indirect link), was possibly similar in all students. Nevertheless, further studies are warranted given the mixed findings of gender differences in SWB indicators in the literature, with some reporting differences (Jiang et al., 2013; Tian et al., 2015b; Liu et al., 2016) and others not (Huebner et al., 2001; Long et al., 2012). Studies have also reported mixed findings of gender invariance in needs satisfaction (Liu and Chung, 2014; Tian et al., 2014b) versus gender differences, particularly regarding higher competence satisfaction in boys (Leversen et al., 2012; Tian et al., 2014a).

Several limitations of this study should be noted. First, the cross-sectional design of this study precludes causal inferences. Future studies should include a longitudinal design to test reverse causation and help establish the sequence of change in the variables. Second, although self-reports are often used to assess *subjective* thoughts and emotional experiences, additional sources are recommended to obtain outcome data on overt SCSs. Future studies could include teacher, parent or peer reports, to provide valuable additional information. Such a methodology could help account for the social desirability bias that can influence students’ responses. Third, the extent to which school curricula are actually designed to facilitate basic needs satisfaction could be examined through document analyses and teachers’ reports.

Despite these limitations, the study has a number of practical implications. The results suggest that educators and parents should consider employing methods to enhance students’ SWB in school by using interventions designed to cultivate their SCS. These could include cognitive-behavioral strategies such as positive reappraisal, cognitive restructuring, using self-talk, and planning steps toward achieving academic as well as adaptive personal and interpersonal goals (e.g., Rosenbaum and Ronen, 2013; Azoulay and Orkibi, 2015). Furthermore, increased perceived needs satisfaction may serve as a “mechanism of change” that may further promote SWB in schools (Shirk et al., 2013). Thus, educators, administrators and policy makers would do well to consider applying classroom practices that support students’ satisfaction of autonomy (e.g., increase choice and provide structured guidance), relatedness (e.g., show kindness and respect, acknowledge students’ feelings), and competence (e.g., communicate expectations, enable active participation in adequate challenges, provide positive and informational feedback) (Niemiec and Ryan, 2009; Doll et al., 2014; Chang et al., 2017) not only to increase academic outcomes but also, as our findings suggest, to increase students’ SWB in school.

## Author Contribution

HO and TR designed the study, analyzed data, and wrote the paper.

## Conflict of Interest Statement

The authors declare that the research was conducted in the absence of any commercial or financial relationships that could be construed as a potential conflict of interest.
